# An alternative to the black box: Strategy learning

**DOI:** 10.1371/journal.pone.0264485

**Published:** 2022-03-18

**Authors:** Simon Taub, Oleg S. Pianykh

**Affiliations:** 1 Department of Computer Science, University of California, Los Angeles, CA, United States of America; 2 Department of Radiology, Massachusetts General Hospital, Harvard Medical School, Boston, MA, United States of America; Universidad de Guadalajara, MEXICO

## Abstract

In virtually any practical field or application, discovering and implementing near-optimal decision strategies is essential for achieving desired outcomes. Workflow planning is one of the most common and important problems of this kind, as sub-optimal decision-making may create bottlenecks and delays that decrease efficiency and increase costs. Recently, machine learning has been used to attack this problem, but unfortunately, most proposed solutions are “black box” algorithms with underlying logic unclear to humans. This makes them hard to implement and impossible to trust, significantly limiting their practical use. In this work, we propose an alternative approach: using machine learning to generate optimal, comprehensible strategies which can be understood and used by humans directly. Through three common decision-making problems found in scheduling, we demonstrate the implementation and feasibility of this approach, as well as its great potential to attain near-optimal results.

## 1. Introduction

The need for optimal decision-making can be seen in many applied problems, ranging from operational planning in manufacturing to risk factor management in healthcare and finance [1–4]. However, in most industries the decision process is still done manually and without optimal design, and is further aggravated by two principal challenges:

- *Process Complexity*: Decisions, processors and resources with different properties can be assigned to each other in many different ways, leading to an exponential number of possible combinations.
- *Variability and Randomness*: Natural deviations and random events (such as emergencies and delays) may disrupt the optimal decision logic, creating long-term bottlenecks and implementation failures.

This makes many decision-rule-learning problems NP-hard, and their solutions–nontrivial. As a result, complex optimization techniques have been intensively studied for decades, and many different algorithms have been developed to find effective solutions. For example, linear programming [5, 6] can be used to create globally-optimal solutions if their cost and constraint functions are linear, while genetic programming [7, 8] and simulated annealing [9, 10] may not attain globally-optimal results, but can work with non-linear problems.

More recently, machine learning (ML) has also been explored to develop even more efficient rule-learning solvers. Common ML approaches include reinforcement learning [11–17], in which one or more agents are trained to make task management decisions (both pro-actively and reactively), and dispatching rules [18, 19], which are often trained with genetic algorithms to assign priorities to upcoming tasks, typically in real time [20, 21].

However, even though each of these pre-ML and ML approaches have yielded remarkable results, they share one fundamental limitation: using complex and humanly incomprehensible *“black boxes”* (BB), to produce *“optimal instance”* (OI) solutions. While this approach is welcome in many areas of machine learning, it runs into several serious problems when applied to real-life decision-making:

- *Accountability* is necessary for many fields where both the solution and the logic behind it needs to be fully understood and trusted. This is particularly true for industries such as healthcare, where mistakes can be fatal [22, 23] and training data can be partial or biased (a flaw that often goes undetected by BBs [12, 24, 25]).
- *BB implementation and maintenance can be expensive*, as they require extensive model (re)training and complex data interfaces, both of which may not be feasible under many real-world constraints. For example, integrating a clinical schedule optimizer or patient risk classifier with the entire electronic patient record data, in real time, presents a serious challenge on its own. Thus, the cost of BBs–and AI solutions in general–often becomes prohibitive in dynamic and time-constrained projects, where constant changes require frequent solution retraining.
- *“Optimal instance” solutions (OIs) cannot be updated* without rerunning them through the same expensive BB cycle. For example, a BB solution cannot be changed by a human to accommodate new or unexpected information because the BB decision logic is too complex and unknown. This renders OIs impractical in most operational environments, where adaptability and speed are imperative.
- Although interpretable models have been used in decision planning before, their primary focus was on identifying certain attributes of the problem, such as the probability of patient no-shows and or clinical risks, rather than creating strategies that help make optimal decisions proactively [26–29].

As a result of these challenges, black box “optimal instance” (BB-OI) solvers are hard to integrate with operational environments, where practitioners frequently need to make critical decisions under severe time constraints. Moreover, precomputed OIs provide no information to guide improvements; they can’t be generalized or converted to human knowledge. It is evident that a different framework is required, one that is flexible enough to survive in a dynamic environment, and interpretable enough to be used by humans.

Therefore, in this work we would like to propose a new approach, which replaces rigid black box incomprehensible solution instances with *optimal comprehensible strategies* (OCS). By OCS we understand a set of rules, satisfying two major criteria:

- *Humanly-comprehensible*, which means that the rules can be easily remembered and used by an average human. As such, the rules should be short, few, and avoiding any complex expressions (therefore reduced to Boolean logic as much as possible).
- *Optimal*, meaning that for a given rule size or complexity limit (such as a set of Boolean decisions), the rule should produce the best possible result.

To discover the optimal rules, we propose to convert the classical “data fitting” ML problem into a new format of a decision-logic learning ML problem, and then solve this problem with an exhaustive (i.e., globally-optimal) algorithm. The latter, although associated with exponential complexity, becomes possible because we are intentionally searching for small-sized decision rules to ensure comprehensibility. As a result, we learn OCS from the data in a similar fashion to humans, by converting multiple observations into experience, but we do this much faster by harnessing the computational power of ML. Finally, we demonstrate the practical advantages of OCS over the old black box solutions, which include an increase in OCS accuracy, adaptivity, and resistance to noise.

In this work, we illustrate our results with scheduling problems, as one of the most typical and widespread examples of non-trivial decision making. However, the same exact approach can be applied to any type of decision-making problem, where historical data on decision outcomes is available or can be simulated.

## 2. Methods and materials

Optimal scheduling is one of the most common challenges in practical application execution. One example is patient appointment scheduling, which is known as one of the most inefficient and expensive parts of the United States’ healthcare system [30, 31]. To study the applicability of OCS “strategy learning,” we used our experience in healthcare scheduling to consider three very common types of scheduling problems, in which diverse tasks (jobs) need to be optimally assigned to a few standard time slots. Matters are further complicated by the presence of noise (e.g., random deviations from expected task durations). As a result, while each problem statement is easy to understand, their OIs are difficult to compute, especially in the presence of noise. Instead, we want to solve these problems by discovering their OCS rules, which eliminate the need for the black box AI setups, and enable humans to make optimal decisions “on the fly”.

The three principal classes of scheduling problems we considered are:

- *Problem P1*: Scheduling *s* short tasks (such as 30 minutes) and *l* long tasks (such as 60 minutes) into uniform timeslots (for example, 45-minute slots). Note that some tasks may take longer than their slots (such as long tasks in our example), which is commonplace in many scheduling problems, and creates the need for optimization. In the noise-free case, the optimal solution to P1 is fairly intuitive: alternate between short and long tasks, to remain on schedule as much as possible, and to minimize wait time. Therefore, we use P1 to compare the OCS discovered by our approach to this intuitively optimal strategy.
- *Problem P2*: Based on P1, this problem adds a time buffer (penalty) for switching between different job types. One common example in real life is the time required to reset a device for performing a different task: for instance, change an MRI scanner coil for a different body part scan. The buffer incentivizes *batch scheduling*, when same-type jobs are put together to save time. This makes P2 more challenging because it subverts the intuitive task-alternating strategy of P1. It also forces the rule-searching algorithm to perform a priority calculation on a scale already unattainable for humans.
- *Problem P3*: Making P2 even more realistic and complicated, P3 should provide up to two 60-minute breaks (one real-life example is mealtimes). Additionally, up to 15 minutes of each break time can be used to finish the current task if it runs late–resulting in a certain “break elasticity,” which is very common in real life scenarios.

With all three problems, we want to avoid the rigid BB schedule solvers, replacing them with humanly-comprehensible strategies. These strategies should answer the main question: given all we know about already scheduled tasks, which task should we schedule next?

### 2.1. Feature selection

As mentioned earlier, we define an OCS strategy as a set of *logical rules*, which prescribe the next optimal decision (*step*, *move*) based on currently known information. Therefore, we need to create a set of features to express this information in the way most manageable for humans. To do so, we defined a variety of features, which can be easily observed and “computed” by a human decision-maker in real operational environments. Table 1 describes the main feature classes we used.

**Table 1:** Main feature classes used to train the OCS algorithm for task-scheduling problems.

Feature Class	Description
Task Processing Progress	Features that reflect current progress in schedule creation, such as current “Step” number, which represents the number of tasks that have been processed so far.
Measures of Delay	Features that describe current, recent, and imminent delays in task processing. Examples include “Currently delayed”, which represents whether or not the schedule is currently delayed, and “Significantly Delayed,” meaning that the schedule will remain delayed for any choice of the next step (job).
Previous Steps	Features that describe the most recent jobs completed, such as “Previous job is…” and “Second-to-Previous job is…” Similarly, many other feature classes also use the “second” descriptor to add additional depth to the information they give.
Remaining Steps	Features which describe the jobs that are left to be scheduled, such as “Is a break available”. Note that such features don’t account for unexpected events.
Comparative Features	Features that involve comparisons of other classes and their values, such as “Previous job is longer than / shorter than / equal to Second-to-Previous job”.

### 2.2. Schedule cost function

In our work we followed the most common definition of scheduling cost [32]. Every schedule instance is comprised of *n* sequential tasks (or jobs). Each job *j* is assumed to have a certain set of known properties, such as estimated (expected) duration time *d*_*j*_, and true (observed) completion time *tj*.

The cost of a scheduled task sequence can be then evaluated with several standard choices of cost functions:

- *Makespan C*_*max*_: The total expected time a schedule requires to be executed [33]:

$$C_{max}=\sum_{j=1}^{n}t_{j}$$

- *Average Tardiness C*_*tardiness*_: The average idle time produced by a given schedule instance:

$$C_{tardiness}=\frac{1}{n}\sum_{j=1}^{n}max\;(0,d_{j}-t_{j})$$

*C*_*tardines*_ represents server inefficiency, as longer idle times result in higher staffing and machinery costs.

- *Maximum lateness C*_*late*_: The longest delay produced by a given schedule instance:

$$C_{late}=\underset{1\leq j\leq n}{\max}{(d}_{j}-t_{j})$$

*C*_*late*_ represents client dissatisfaction, as longer delays waste their time and may create bottlenecks that they are unwilling or unable to tolerate.

In practical applications, it is particularly important to increase job timeliness without introducing significant idling (for example, to improve client satisfaction without crippling server efficiency). Therefore, we focus on minimizing both *C*_*tardiness*_ and *C*_*late*_ by minimizing their combined cost:

$$C_{\alpha }=\alpha C_{tardiness}+(1-\alpha )C_{late},0<\alpha <1$$

The weight coefficient α defaults to 0.5, but we will briefly discuss the effects of changing α in the results section.

### 2.3. Rule-learning scheduling problem formulation

The main goal of our approach was to re-formulate the classical scheduling problem (finding the optimal schedule instance OI) into a *rule-learning* scheduling problem (discovering *optimal comprehensible rules* to build optimal instances OI). To do so, we modeled human scheduling behavior, observing *k* already scheduled tasks, and calculating which task should be scheduled next to minimize the overall schedule cost.

**Fig 1:**
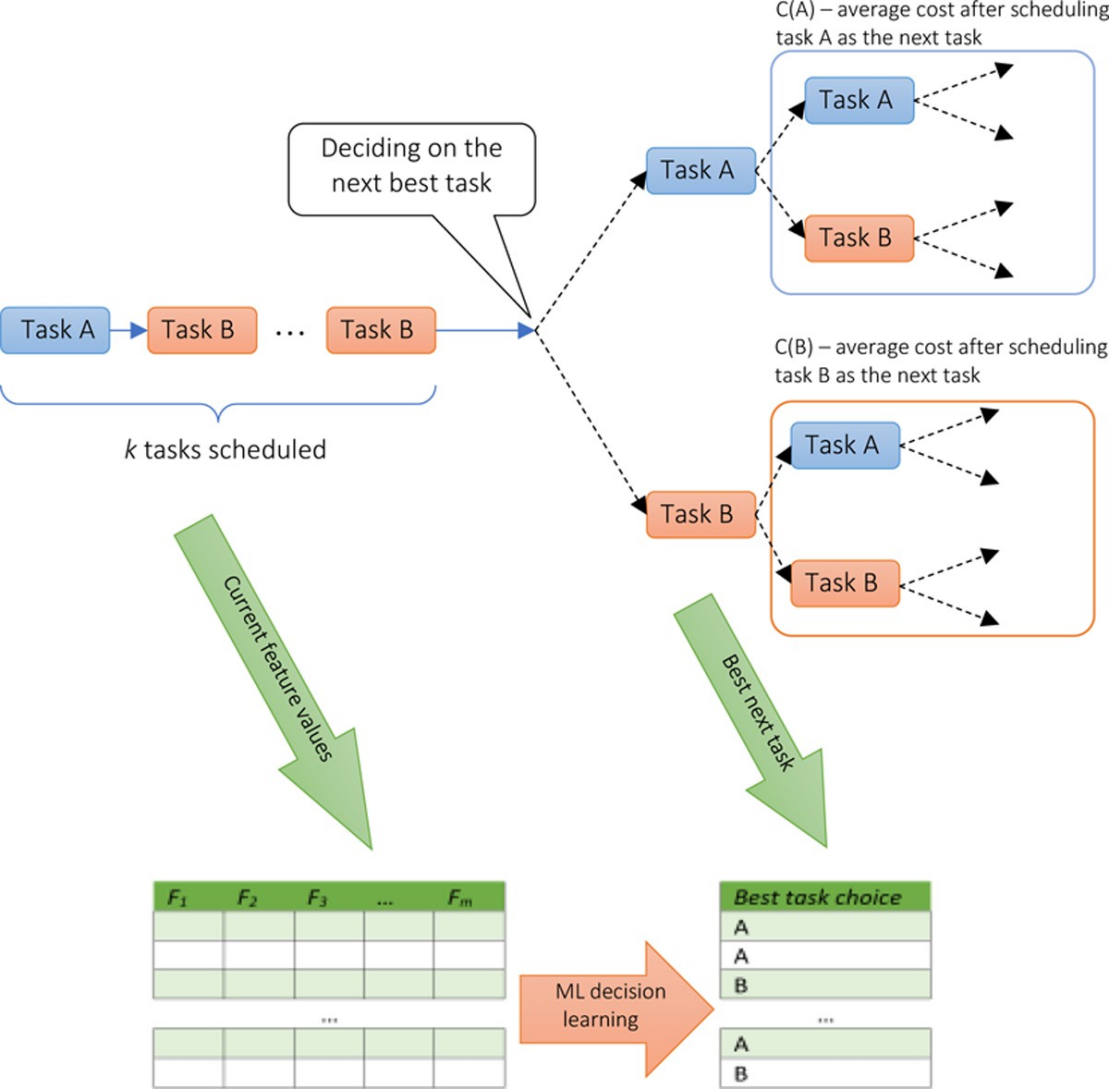
Converting scheduling problem into the optimal decision-making ML classification problem.

We illustrate this approach in Fig 1, where a scheduling decision-maker, after making the first *k* scheduling assignments, needs to decide between two possible task types, A and B, to be scheduled as the next (*k*+1)^st^ task, to minimize the predefined cost function. To do so, we need to consider all possible branching “tails” of remaining tasks, and compute the average cost C associated with each choice. The task with the lowest-cost tail is then prescribed as the next to be scheduled (“best decision”). Associating this best next step with the information (features) *F* observed from *k* already scheduled tasks creates a map from the feature space to the best task choice. Consequently, “learning” this association with ML rule model discovers the best OCS rules to predict the next best task selection as accurately as possible.

### 2.4. Model selection

**Fig 2:**
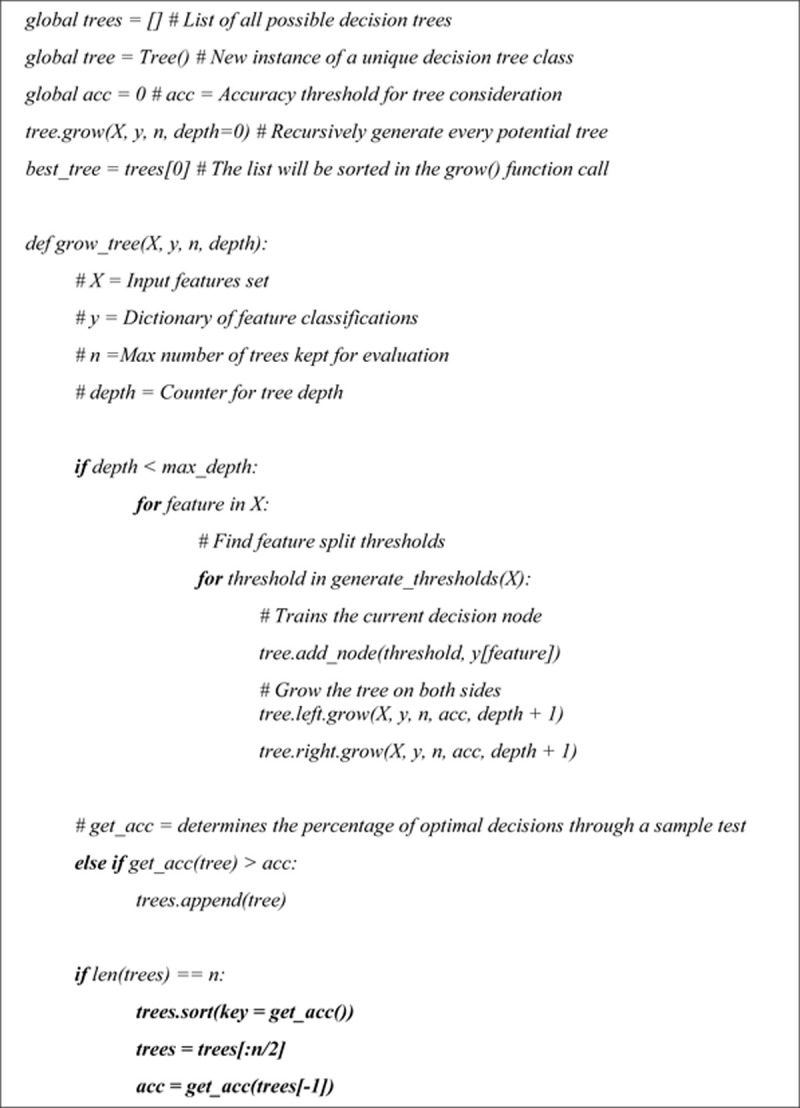
Pseudo code describing the process of generating an exhaustively optimal decision tree. All possible decision trees of a small size are generated, to select the most accurate tree.

We used the following ML models to learn decision logic from the data described above:

- *M1*: *Exhaustive Decision Tree*, which finds the globally-optimal tree by exhaustively searching through all possible trees with the same number of leaves (Fig 2). The advantage of this model is that it always learns the best possible decision strategy. The disadvantage is the high computational cost.
- *M2*: *Exhaustive Binary Rule Learner*, where, similarly to M1, the model exhaustively learns the best Boolean form of a given complexity (defined as the total number of conjunctions and disjunctions). Thus, M2 also finds globally-optimal strategies, and fits better into human-like decision making (“to do or not to do” a certain task); it is also faster than an exhaustive tree in M1. The disadvantage of M2 is that it can only be used in binary outcomes.

With no exhaustive solvers readily available, we developed our own versions for the two models, M1 and M2. Note that even though time-consuming exhaustive learning is very unattractive in general ML problems, it becomes possible and important in OCS learning, where we need to find only the *smallest* models (rules) to be easily understood and remembered by the humans.

### 2.5. Constraints on interpretability

Our definition of OCS strategies relies on the concept of humanly-comprehensible decision making, extensively studied in human phycology. According to this research, humans can only process problems with up to four variables [34] before their underlying logic becomes incomprehensible. As a result, a decision tree for OCS strategy representation must be limited by three variables past the root node.

It has also been demonstrated that humans are more efficient when dealing with a few discrete choices rather than continuous variables [35, 36]. Therefore, we intentionally limited most of our decision features to simple Boolean expressions, such as “is current job delayed?” instead of “how much is the current job delayed?” (Table 1). In the case of continuous variables such as “step,” we categorized them by setting simple thresholds. For example, “step > *n* / 2 jobs” became “more than 50% of jobs scheduled” (“more than half of steps made”).

## 3. Results and discussion

In this section, we present the principal “strategy learning” results for the three classical scheduling problems stated above. For each problem, we created the training set by considering all possible scheduling sequences, and determining the next optimal task at every step within each sequence (such that it minimized the overall cost). Then we use globally-optimal ML classification learner to discover the best strategy as predicting the optimal task choice (Fig 1). Additionally, based on our experience with the real scheduling data [37], we used 5% multiplicative noise to distort the observed task durations *t*_*j*_.

### 3.1. Problem P1: Mix of long and short tasks

**Fig 3:**
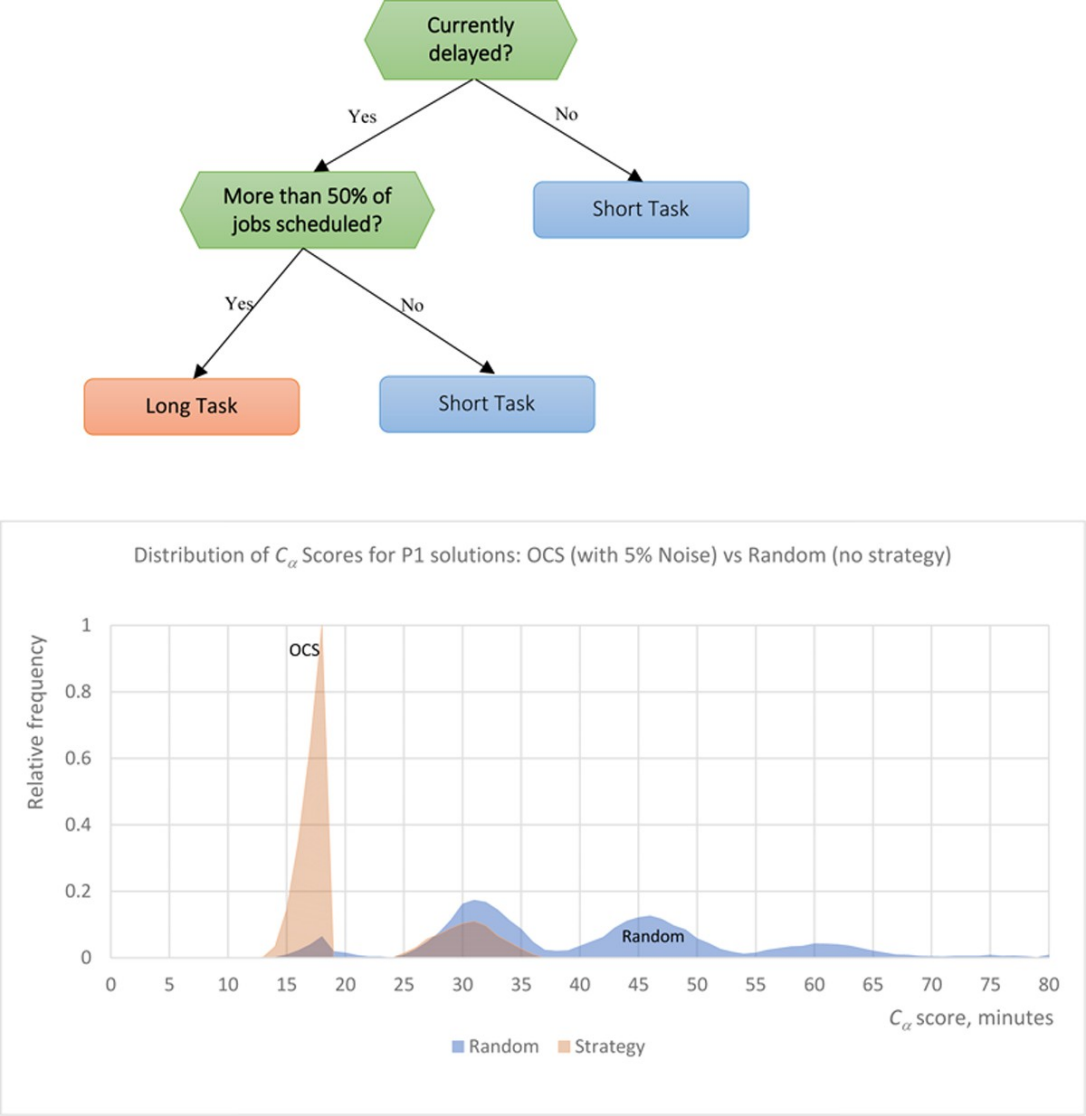
(Top) A simple OCS strategy learned by the ML model for problem P1 with two different job sizes. (Bottom) Strategy efficiency histogram, comparing the cost C_α_ of OCS strategy (represented by the decision tree) to random “strategy-less” scheduling.

The decision tree in Fig 3 (top) displays the optimal decision-making strategy, discovered by our exhaustive-tree ML algorithm M1, applied to the baseline problem P1 (scheduling of a mix of long and short tasks). Interestingly enough, the strategy corresponds to the intuitive solution of alternating the tasks depending on the current delay, but suggests a more adaptive way of implementing it, and adds one more optimizing decision split. Thus, the root node is not “Previous Job is Short” (which may have been expected given the intuitively best strategy of alternating between short and long tasks), but “Currently Delayed.” In this way the optimal strategy learns to work with a variety of suboptimal schedules, where previous tasks might have been scheduled in non-optimal ways (e.g., two long tasks instead of alternating long and short). Such schedules can only be corrected through the “Currently Delayed” feature, which focuses on constantly minimizing delay instead of simply alternating.

The second decision split is even more interesting, as it looks at whether more than half of the tasks were scheduled already. This less trivial optimization ensures that long jobs, which disrupt the schedule the most, are typically scheduled for the end of a workday, where their impact on the remaining schedule is minimal. This addition optimization reduced costs by 8%.

To evaluate the overall efficiency of the OCS strategy, we computed a “strategy efficiency histogram”, showing the probability distribution of each schedule cost *C*_*α*_ for two principal scenarios: using our OCS strategy represented by the optimal decision tree, and using random “strategy-less” scheduling. As one can observe in Fig 3 (bottom), the OCS strategy achieves significantly better performance: the average OCS schedule cost corresponds to the near-optimal 93^rd^ percentile of the overall schedule cost distribution (we will further refer to this metric as “percentile efficacy”). This means that a human armed with simple and interpretable OCS decision logic can create highly optimal schedules even in the presence of noisy data and suboptimal task assignments. This also means that even the most complex BB solution will improve this simple OCS strategy only marginally, but at a very high cost.

Since P1 problem was deciding between only two tasks, we also used our exhaustive search binary model M2 to discover the best Boolean expressions for P1 scheduling rules. The most powerful strategies learned by the model were to schedule short jobs when (“Currently Delayed” and “Less Time Spent than Left) and (“Currently Delayed” and “More than 75% of Jobs Scheduled) rules are met, thus essentially reproducing the logic of the optimal decision tree M1. Moreover, further increasing the number of variables included in each binary rule did not yield significantly better results: the average instance percentile for the best combination of three features was still in the 93^rd^ percentile, indicating that the two-feature OCS strategy is nearly optimal.

### 3.2. Problem P2: Mix of long and short tasks with batching

**Fig 4:**
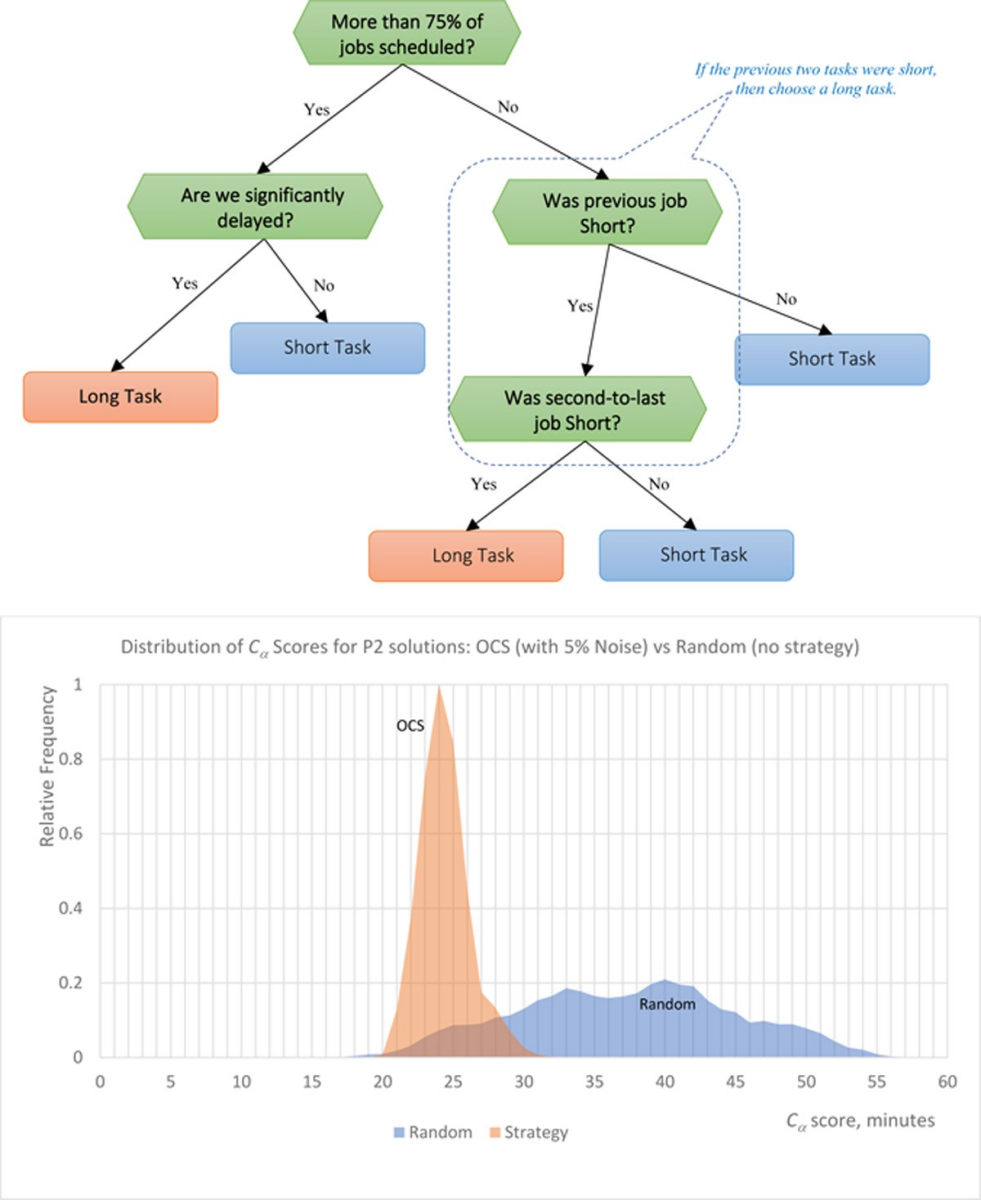
(Top) OCS strategy learned by M1 for the problem P2 with two different job types and a penalty for switching between job types. (Bottom) Performance of the OCS strategy compared to the random (no-strategy) scheduling.

The optimal comprehensible strategy discovered for to the second scheduling problem P2 is shown in Fig 4. The strategy is more complex than the baseline P1 solution, largely because the problem itself is much less intuitive. The need for the buffer necessary for changing equipment between different tasks subverts the most obvious task-alternating strategy as significantly increasing the delay. To find a better strategy, the algorithm suggests alternating between couples of short tasks and then long tasks (e.g. [long] then [short, short] then [long] again–see the right branch of the decision tree). This cuts down on the delay caused by alternating between jobs while still trying to keep the overall schedule on time, and one can simplify this logic further to: “if the previous two tasks were short, then choose a long job. Otherwise, choose a short job.” In addition, the threshold for defaulting to long tasks has been extended from “More than 50%” of Jobs Scheduled to “More than 75%,” primarily because, with the incentive to batch similar jobs together, the risk for generating extreme delays is higher. Running our exhaustive Boolean rule model M2 confirmed the same strategy as identified by the exhaustive tree model M1.

One may fear that same-task batching, required in the optimal P2 solutions, might make it more sensitive to noise. Because the threshold for defaulting to long tasks increases from “more than 50% of jobs” scheduled to “more than 75%,” there is a higher risk that unexpected events will force the scheduler to start the day with many more short jobs than long ones, potentially leading to large bottlenecks at the end of the day. However, despite this risk, the OCS strategy discovered for this problem results in a very efficient 95th-percentile solution even when significant noise is present, thus resulting in a very robust and highly optimal decision rules.

### 3.3. Problem P3: Mix of long and short tasks with breaks

**Fig 5:**
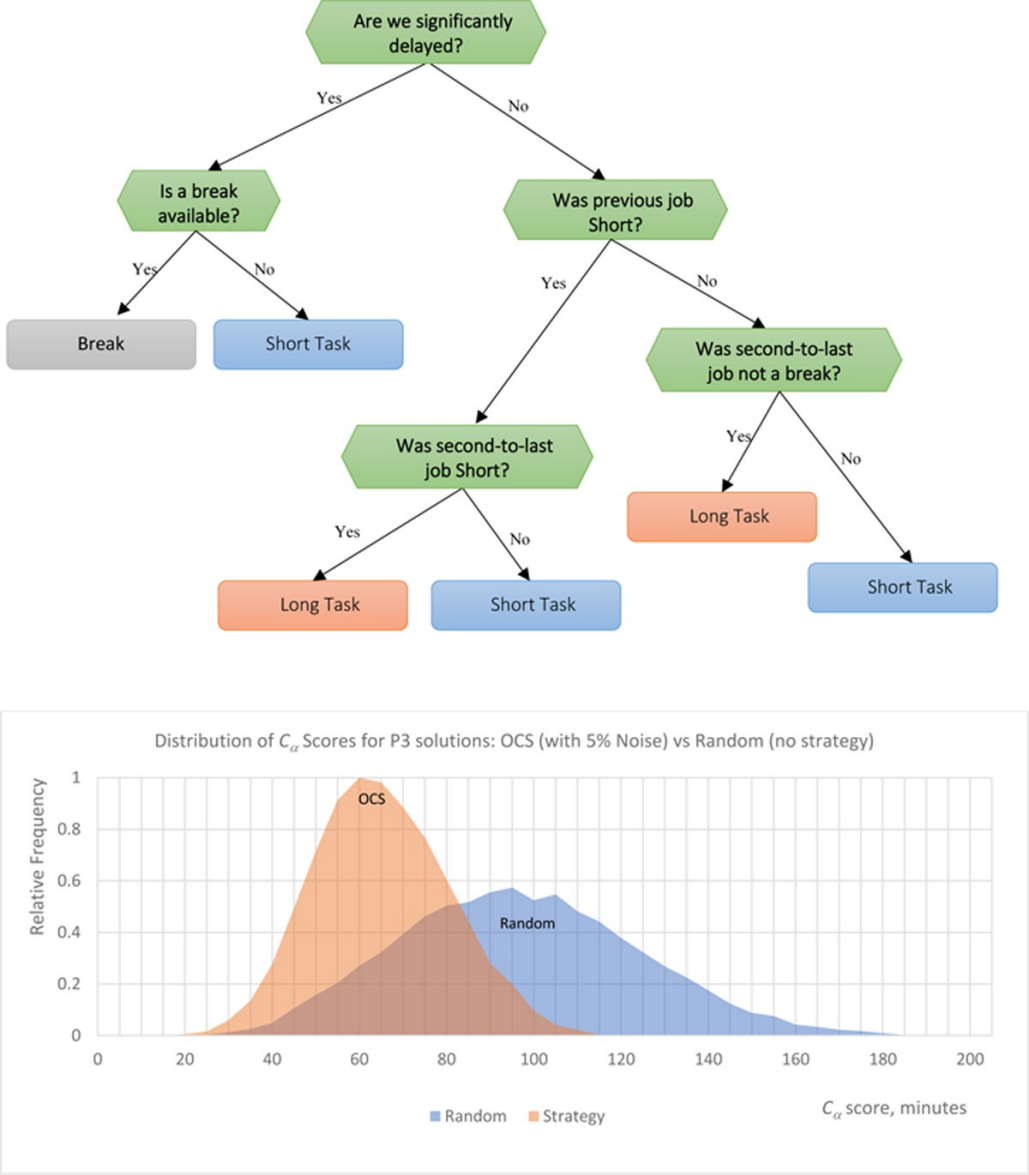
(Top) OCS strategy learned for the problem P3 with three different job sizes and a penalty for switching between task types. (Bottom) Performance of the OCS strategy compared to the random (no-strategy) scheduling.

The optimal OCS strategy for the 3-job “brake scheduling” problem P3 is even less trivial, but surprisingly effective (Fig 5). Similar to P1, the root node for the optimal strategy solution refers to whether the schedule is significantly delayed (Fig 5, top): if a break is available, then it can be used to minimize the current delay and potentially change equipment (which includes a buffer) without increasing the delay further. Otherwise, the algorithm follows the previously shown double-alternating strategy ([long, long], [short, short]), with the feature “Second-to-Previous Job is Not a Break” used to distinguish between long jobs and breaks (instead of the more simple “Previous Job is a Long Job”). Interestingly, setting α to 0.75 instead of 0.5 in the *C*_a_ equation (to favor the idle time minimization) has a significantly bigger impact on this problem than others, decreasing efficacy significantly, from 91% to 82%. This is a much larger shift than in any other problems; this may occur because prioritizing the reduction of idle time is much more difficult when workers require breaks. Conversely, changing α to 0.25 increases strategy efficacy beyond the 91^st^ percentile, because it allows algorithm to absorb delays using a natural “break elasticity” defined in the description of P3.

Even in the presence of 5% noise, schedules generated by the OCS P3 strategy fall, on average, in the 91^st^-best percentile of all possible instances, thus still resulting in highly optimal solutions. This confirms, once again, how ML can be used to discover very efficient, concise, and comprehensible decision rules, which can be then used by humans to make optimal decisions without “black boxes”.

### 3.4. The effect of noise

One of the principal benefits of optimal strategy learning, in comparison with the current optimal instance learning, lies in its ability to remain applicable to the suboptimal scenarios, when the schedule execution may differ from the prescribed. Shorter rules provide a better generalization of what needs to be done to achieve the best goal; and this generalization makes them more resistant to disruptions.

**Fig 6:**
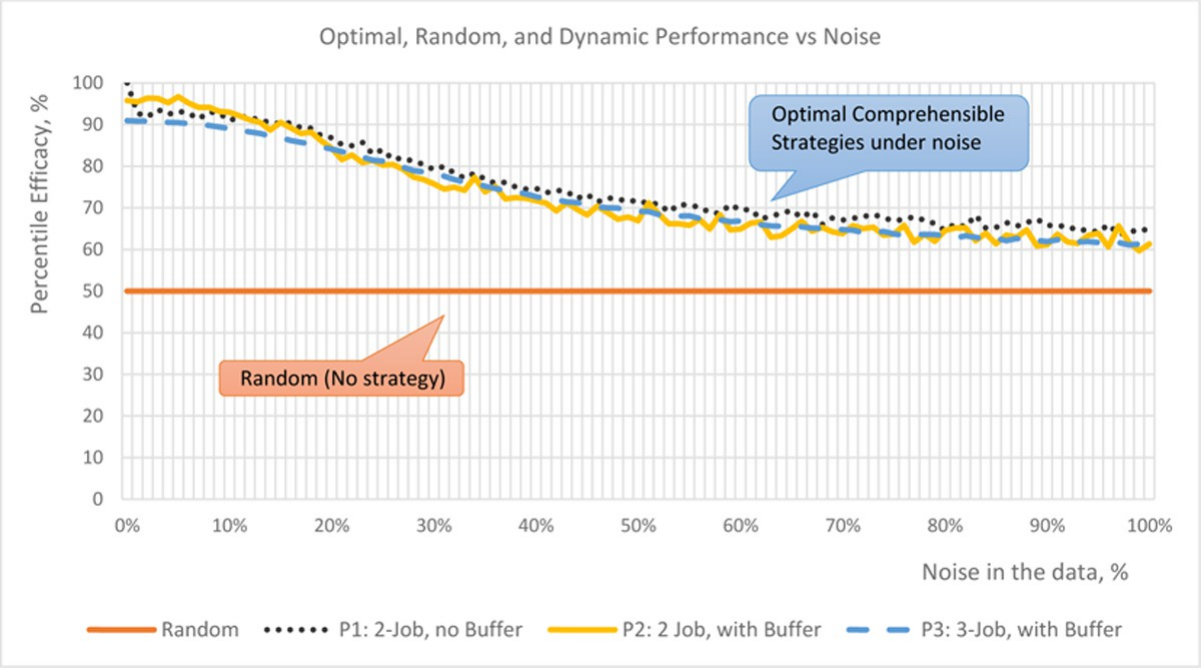
The performance of optimal strategies under various levels of noise in the underlying data. Percentile efficacy represents the performance of a given schedule instance relative to all possible instances.

We illustrate this by studying the effects of noise–as variability in the actual task execution time–as one of the principal sources of real-life schedule disruption. Fig 6 shows the performance of optimal strategies under various levels of noise in the underlying data (horizontal axis). Percentile efficacy (vertical axis) represents the performance of a given schedule instance relative to all possible instances; thus, an instance with a percentile efficacy of 73% has cost better than 73% of possible schedules, while randomly generated schedule corresponds to 50%.

As one can see, all three optimal strategy models continue to produce significant gains even when exposed to massive 100% noise. This resistance to noise is likely caused by the fact that the OCS strategies were trained on noisy data. The ability to incorporate such noise into the OCS model training represents another advantage of our approach. On one side, adding various noisy samples significantly increases the number of training records, and obscures the optimal processing strategy, making it impossible for the human observers to discern. But on the other side, this challenge can be perfectly handled by ML learning algorithms. By delegating optimal strategy extraction to the computationally efficient models, we eliminate human trial and error, and the time it takes to arrive at a similar result.

### 3.5. The benefits of globally-optimal ML

As described previously, the OCS “comprehensibility” requirement justified the use of exhaustive (globally-optimal) ML models M1 and M2, instead of more conventional, but suboptimal. “greedy” ML. To evaluate the benefits of this approach, we compared our exhaustive tree-based model M1 to the standard “greedy” tree classifier algorithm (CART decision tree classifier as implemented in Python [38]):

As one can observe from Table 2, the exhaustive models visibly outperform the greedy, and this becomes more pronounced as problem complexity increases. Thus, learning the “best possible” OCS rules can still be done efficiently when the model size is small, resulting in significant gains in decision logic quality.

**Table 2:** The relative performance of the CART and exhaustive scheduling algorithms.

Problem	Exhaustive tree optimization M1	Greedy tree optimization
P1	**93%**	91%
P2	**95%**	87%
P3	**91%**	81%

## 4. Conclusions

The main goal of our work was to demonstrate that in decision-making problems, one can efficiently replace complex, uninterpretable, and hard to maintain “black box” solutions with a different type of machine learning: Optimal Comprehensible Strategies (OCS). These strategies help us achieve several principal goals:

- Discover decision rules that can be easily understood and used by humans.
- Learn from the data in a human-like way, yet completely eliminating expensive and risky human trial-and-error.
- Create stable solutions in the presence of most typical decision disruptors, such as noise or suboptimal choices.
- Achieve significant decision quality gains at the minimal possible effort.

We demonstrated these properties through a case study with three scheduling problems, although our OCS approach is completely general and can be applied to other areas requiring human decision-making (such as healthcare, manufacturing, finance, game theory). We also explored the natural connection between the OCS and globally-optimal ML models, when exhaustive searches become very affordable as the model size is reduced.

Although we did not directly compare the effectiveness of BBs and supervised models in this work, we built upon previous research that had already outlined the trade-off between the accuracy and interpretability of machine learning models [22, 39]. It is also important to note that the OCS considered in our work have produced solutions with higher that 90^th^ quality percentiles. As a result, at least in our case study, even if further improvements through BBs are possible, they will be minor, and will hardly justify the loss of comprehensibility, lack of adaptiveness, and high implementation cost of black boxes.

Thus, we find these results very encouraging, both numerically and conceptually, with great potential to improve many real-life applications. We firmly believe that using AI to discover the optimal comprehensible strategies should eliminate expensive human trial-and-error, and provide the best example of human-computer interaction. OCS can open a new way to convert data analysis and machine learning into the humanly-interpretable knowledge, which can be directly applied, understood, and refined to advance our understanding of the world around us.
