# Correction: An alternative to the black box: Strategy learning

**DOI:** 10.1371/journal.pone.0270441

**Published:** 2022-06-21

**Authors:** Simon Traub, Oleg S. Pianykh

The first author’s name is spelled incorrectly. The correct name is: Simon Traub. The correct citation is: Traub S, Pianykh OS (2022) An alternative to the black box: Strategy learning. PLoS ONE 17(3): e0264485. https://doi.org/10.1371/journal.pone.0264485

## References

[1] Taub S, Pianykh OS (2022) An alternative to the black box: Strategy learning. PLoS ONE 17(3): e0264485. 10.1371/journal.pone.0264485 35302996 PMC8932616

